# Integration of health solutions into government systems: a tool for assessing readiness **

**DOI:** 10.12688/gatesopenres.13193.1

**Published:** 2020-11-19

**Authors:** Emily Lawrence, Amanda Pain, Jessica Crawford, Kim Baker, Ruth Bechtel, Aida L. Coelho, Alvo Ofumane, Joseph Roussel, Bvudzai Magadzire

**Affiliations:** 1 VillageReach, Seattle, WA, 98102, USA; 2 VillageReach, Maputo, Mozambique

**Keywords:** transition, sustainability, government ownership, solution adoption, institutionalization, scale-up

## Abstract

Government partnerships are essential for many health solutions to sustain impact at scale, particularly in low-resource settings where strengthening health systems is critical for Universal Health Coverage. Many non-governmental organizations (NGOs) and funders ultimately want solutions to be integrated into public health systems by transitioning solution ownership, management and/or operation to government. However, NGOs and their government partners have limited guidance on how to effectively determine when a solution is ready to transition in a way that will maintain impact long term. To address this need, VillageReach developed the Transition Readiness Assessment (TRA) based on our transition to government theoretical framework. The framework was developed to define both factors related to a solution, as well as external influences that affect a solution’s success.  The framework identifies seven dimensions of solution readiness: the political, economic, and social context; solution design; resource availability; financial management; government strategy; government policy and regulations; and organizational management. The TRA measures those dimensions and assigns each one a readiness score. We developed the framework and TRA for VillageReach solutions, as well as to share with government partners and stakeholders.

This Open Letter outlines the TRA development, details empirical examples from applying the tool on two VillageReach solutions, and presents recommendations based on our lessons learned. Stakeholders working to transition solutions to government can utilize both the TRA and our lessons.

## Introduction

For many non-governmental organizations (NGOs), transitioning ^a^ a solution to government ownership, management, and/or operation is the best way to sustain solution impact at scale. Government partnerships contribute to solution design and implementation through networks and infrastructure to reach more people, fiduciary authority over spending, and an understanding of their population’s needs and values ^1, 2^. Transitioning effective solutions to government can support stronger, more sustainable public health systems, which are necessary for achieving Universal Health Coverage ^3^, a critical target in Sustainable Development Goal 3.

Large funders, such as the United States Agency for International Development, have shown increased interest in building country self-reliance. This work has primarily centered on capacity building by transitioning large-scale initiatives from a funder to government ^4– 12^. However, for NGOs looking to transition solutions into public health systems there is little guidance on how to transition a solution or assess solution readiness for transition ^3, 8^. There are few examples evaluating how transition of solution ownership affects long-term solution impact ^4^, and NGOs and their government partners lack guidance on collaborative transition planning and on how to adapt to changing solution maturity and context ^2^.

To address this need, VillageReach developed the Transition Readiness Assessment (TRA) based on our transition to government theoretical framework. The TRA helps address the lack of available resources to guide solution transition. We also present two empirical examples of its application on two VillageReach solutions within the same sub-Saharan African country and provide recommendations for future users. 

## Transition Readiness Assessment description

**Figure 1.**
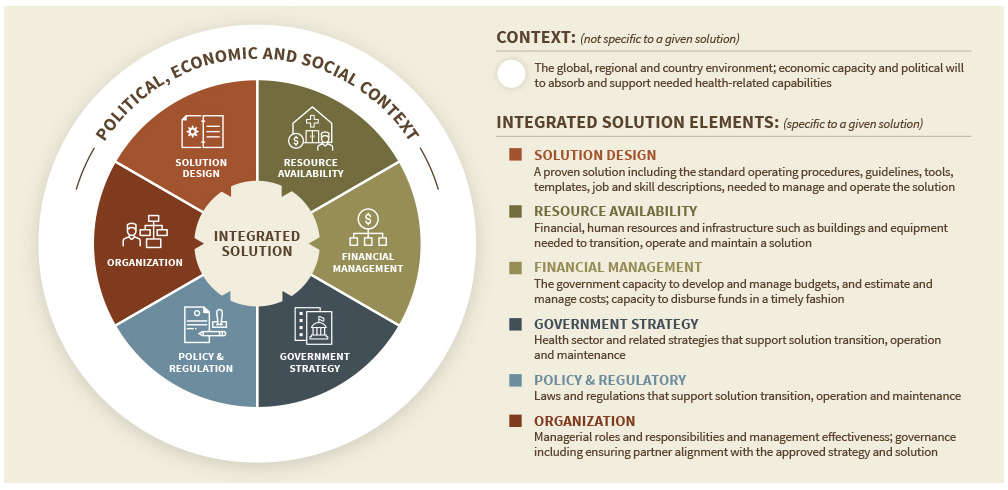
Transition to government theoretical framework.

The TRA was developed based on the transition to government theoretical framework ( Figure 1) ^13^. The framework is based on the concept that successful solutions require an enabling environment, which includes measurable factors both within and external to a solution. We have identified these factors as the seven dimensions of solution readiness, which we define as an indication that a solution is likely to maintain impact post transition. (Here we define a solution as a combination of processes, products, principles, organization, tools, metrics, and collaboration that provides the functionalities needed to solve a defined problem). These dimensions, identified through a literature review and VillageReach’s organizational experience, include: (1) the political, economic, and social context; (2) solution design; (3) resource availability; (4) financial management; (5) government strategy; (6) government policy and regulations; and (7) organizational management ^13^.

The TRA was informed by findings from a scoping review, an analysis of existing sustainability and transition tools, and VillageReach’s organizational experience ^5– 10, 14, 15^. Three VillageReach staff conducted the scoping review by searching both peer-reviewed and grey literature on PubMed, GoogleScholar and Eldis.org. The team used several key words in different combinations such as “transition+to+government,” “sustainability+of+donor+funded+health,” and “Country +ownership + transition +health” to identify articles. From this search, we identified 28 articles for further review, for each article, we noted the author name, journal title, year, region, and focus of paper, dimensions related to transition or sustainability, definitions for each dimension, key results and key takeaways. Existing sustainability and transition tools were identified through this review and through consultations with experts in the field. Identified tools were assessed to understand the most common data collection methods, indicators, and scoring approach each of the tools utilized. For example, all tools had either indicators or categories dedicated to assessing funding stability, presence of a champion and organizational capacity, all of which are reflected in the TRA. This analysis informed the TRA’s scoring approach, format, dimensions, sub-dimensions, guiding questions and indicators. 

The TRA is an Excel™ based tool that measures at a given point in time 49 indicators assigned across the seven dimensions of readiness, each of which also has several sub-dimensions that are measured ( *Extended data* ^16^). Each of the 49 indicators are scored by placing them in one of four categories: (1) fully in place, (2) partially in place, (3) not in place, or (4) not applicable ( Table 1) ^6^. Final solution scores for each dimension and sub-dimension allow NGOs and their government partners to identify transition barriers, and where additional support is needed for transition success.

**Table 1.** TRA scoring key.

SCORING	DEFINITION	EXAMPLE
Fully in place	All aspects of the indicator have been met	List of existing approved cadres for Ministry of Health with funding that adequately covers the needs of the solution
Partially in place	Action has been taken or is in progress, however not all aspects of the indicator have been met	List of approved cadre for Ministry of Health, however funding does not cover staff to meet the needs of the solution
Not in place	No part of this indicator has been met	Ministry of health has not approved a cadre of staff for the solution nor is there secured funding for the positions to meet the needs of the solution
Not applicable	This can mean either this indicator is not valid for this solution or country or that there is insufficient data	The solution doesn’t need staff to manage or operate the solution

## Approach for applying the TRA

In May 2019, VillageReach applied the TRA on two solutions currently transitioning to government within the same sub-Saharan African country. One is a vaccine delivery solution based on VillageReach’s approach to supply chain strengthening (hereafter referred to as solution 1) ^11^. The second is a similar solution that integrates vaccines along with other medicines and uses an outsourced transport provider (hereafter referred to as solution 2) ^12^. Both solutions were developed and implemented in close partnership with government, but solution 1 currently operates at the national and provincial levels, while solution 2 operates at the provincial level only.

We applied the TRA on both solutions using qualitative data collection methods such as document review, key informant interviews, a modified nominal group technique, and a team-based consensus building approach ^17^. This allowed the research and solution management teams (EL, RB, BM, JR, AC, and AL) to agree on final scores for each indicator. The assessment team was led and coordinated by a senior health systems researcher (BM) and supported by a qualitative analyst with a background in health systems research (EL). Other study team members included the solution program staff, who were involved in the development and implementation of the solution and who are currently managing solution transition.

Application of the TRA consisted of six iterative steps following a modified nominal group consensus building technique:

1. *Data gathering and review* – For each solution, two team members independently reviewed all existing data sources related to the 49 indicators for each solution (solution 1: EL and BM; solution 2: JR and AL). Team members reviewed evidence that supported identified indicators and noted indicators where additional data or clarification was needed. Data sources reviewed included monitoring and evaluation reports, national strategy documents, an external evaluation of government accountability and organization, and budgeting guidelines.
2. *Initial scoring* - The same two team members, for each solution, collaboratively assigned initial scores for each indicator. This was done through an in-person half day working meeting to discuss each guiding question and associated indicators in the TRA, then reviewing the evidence identified in step 1 to agree on a score of fully in place, partially in place, not in place, or not applicable. Justification of assigned scores was documented in the comments section of the TRA Excel tool (eTable1). When team members disagreed on a score, or did not feel there was sufficient evidence to apply a score, they noted this in the notes section of the TRA Excel tool for further discussion during step 3.
3. *Team based consensus building* - The same two team members presented the preliminary TRA findings to each of the larger VillageReach solution teams in a two-hour working meeting. During this meeting, initial TRA results were presented facilitating a discussion and revision for final group consensus on each indicator score. Team members provided critical input that was not possible to obtain through data sources, such as current projects and discussions happening with the government, as well as putting key evidence obtained through the document review into the context of transition. Key points from this discussion and any changes to the indicator score were documented in the comments section of the tool. Through this process, the team also identified indicators where government stakeholder perspectives were needed. The team decided that the best way to obtain this input was through key informant interviews.
4. *Obtaining government input through key informant interviews* - The team obtained government perspectives through key informant interviews with national (n=2) and provincial government staff (n=4) working with VillageReach on solution transition. Key informant interviews focused on the guiding questions and on the indicators where government input and data were deemed necessary to apply an accurate readiness score. Interviews were conducted by EL and AO. One interview with a national government key informant was conducted in English over Skype, while the rest were conducted in Portuguese in person. Interviews were not recorded, rather the interviewer filled out a detailed interviewee debrief form ( *Extended data* ^18^) following the interview. The debrief form contained questions to help the interviewer quickly summarize the discussion. EL, AO and BM met following each interview to discuss the interview and determine if additional interviews were needed. The team agreed that we reached saturation in our sample, and no additional interviews were carried out.
5. *Consensus building* ****- Two team members summarized (AO and EL) the key informant interviews by solution and question, and then one team member (EL) reviewed selected indicator scores and revised them to reflect interview findings. Changes to indicator scores were documented in the comments section of the tool. The final scored TRA was then sent via email for final review and validation by each VillageReach solution team.
6. *Quantification of scores* - Lastly, one team member (EL) developed summary scores for each sub-dimension of transition readiness by calculating the proportion of indicators scored as fully in place within each sub-dimension. An indicator was omitted from the denominator of the summary score if it was labeled not applicable.

## Key observations

Overall application of the tool for these two situations was a success ( *Extended data* ^19, 20^). The tool was easy to use, facilitated group discussion and consensus building, and provided an opportunity to document challenges. Assessing the scores across and between solutions ( Table 2) allowed us to pinpoint barriers and enabling factors for solution transition, as well as better understand each solution’s strengths and weaknesses.

**Table 2.** Transition readiness assessment scores by solution.

	Transition Readiness Assessment Dimension	Solution 1 Percent of indicators scored as "fully in place"	Solution 2 Percent of indicators scored as "fully in place"
**Enabling Context**	Political, Economic & Social Context	13%	13%
**Integrated** **Solution**	Solution Design	43%	57%
	Resource Availability	17%	71%
	Financial Management	43%	71%
	Government Strategy	20%	100%
	Policy & Regulatory	Not Applicable *	100%
	Organization Management	10%	64%
	**Average Total Score** **acros sdimensions**	**24%**	**72%**

*Policy and regulatory factors were scored as not applicable by the solution team as they felt actions in this category would not impact the sustainability of the solution post-transition.

Solution 1 overall scored worse than solution 2. Solution 1 had an average of 24% of indicators across the seven dimensions as fully-in place, while solution 2 averaged 72%. Identifying why these solutions’ scores were so different provided valuable input not only for existing transition plans for solutions 1 and 2, but also for future VillageReach solution transitions. For example, solution 1 had low scores in four of the seven dimensions: (1) Organizational Management, due to poor accountability for improving solution performance at the provincial level; (2) Resource Availability, due to late disbursement of donor funds to the national government, as well as lack of maintained vehicles for supply distributions; (3) Government Strategy, because provincial champions lacked guidance on how to advocate at the national level post-transition; and (4) Solution Design, because despite standard operating procedures, no comprehensive toolkit detailed how to manage, operate and adapt the solution long term. The TRA results from solution 1 also demonstrated the importance of continually adapting solutions in response to shifting government priorities and supply chain strategies. For example, solution 1 was aligned with government priorities at implementation, but over time government priorities evolved, affecting the solution’s impact and sustainability.

Solution 2 scores helped us identify effective processes for transition, but also demonstrated the value of examining sub-dimension scores closely. For example, solution 2 scored high on four dimensions: (1) Solution Design, because a toolkit was developed that provided government staff with essential operational details; (2) Organizational Management, because a national level position provides oversight and accountability for provincial level activities; (3) alignment with Government Strategy because of an identified national level government champion, and strong political support to adopt and scale the solution; and (4) Policy and Regulatory dimensions, because the solution aligned with the national government supply chain strategy. Solution 2, however, also had low scores in some important sub-dimensions that required our immediate attention. For example, under Financial Management, the sub-dimension Costing had a low score indicating a need for additional analysis to understand operating costs and how they would evolve as the solution scales. Based on this finding, VillageReach staff realized that we did not have a concrete understanding of provincial financial flows or a true understanding of long-term solution costs.

In summary, by reviewing TRA results we better understood where to apply additional time and resources to help ensure successful solution transition to government ownership.

## Recommendations for future application

Piloting this tool provided important insights for future applications of the TRA.

*First, the TRA should be used in a workshop format.* We initially applied the TRA internally and obtained government input only after assigning initial readiness scores. Administering the tool with all relevant stakeholders in a workshop setting would be more beneficial and allow stakeholders to collectively discuss and build consensus around scoring complex indicators for transition readiness. Representatives from all government levels --national, provincial, state, and local should attend the workshop to establish sustainable support in the event of political changes. 

*Second, detailed user guidance for the tool would help ensure that NGOs and their government partners use the tool accurately and consistently.* VillageReach developed an accompanying guidance document ( *Extended data* ^16^) for the TRA tool after this initial application. The guidance provides users of the tool with information on the TRA development process, and on how the tool helps inform transition planning. The guidance document also defines roles and responsibilities for administering the TRA and provides direction on scoring indicators and interpreting results. 

*Third, the TRA should be used early and often throughout transition.* ****The TRA was developed after the transition process began for both solutions 1 and 2, which gave us limited time for adjusting solution transition plans. While the scores still provided us with useful feedback, ideally stakeholders would apply the TRA tool earlier in the transition process. This will provide stakeholders with adequate time to address all dimensions with low scores and discuss with funders any possible adaptions to timeline or resources. Stakeholders should also consider applying the tool multiple times throughout the transition process to continuously adapt the transition plan to the current political, economic, and social environments. This in turn will improve the solution’s readiness to transition and ensure sustainability long term.

*Fourth, the TRA should be applied in partnership with the government entity adopting the solution.* We gained government feedback after internally assigning scores to each dimension. While this input was utilized to adapt final dimension scores, having a government counterpart to lead or co-facilitate applying the TRA can amplify government voices, and ensure government commitment throughout the transition process. Since the government will ultimately own and operate a solution post transition, working collaboratively with government partners throughout the transition process is critical.

*Fifth, the TRA can be useful post transition to assess solution sustainability and ongoing impact.* To date the TRA tool has been used to inform effective transition planning. In the future we plan to combine the TRA tool with outcome evaluation methods as solutions are fully transitioned to government ownership. This evaluation will measure whether the solution was successfully integrated into government systems and whether the process of transition was effective. The results will help inform future planning for transitioning solutions to government.

## Limitations of the TRA

The TRA is an operational tool to help facilitate discussion. It provides a readiness score at one point in time and does not provide a minimum score required to achieve sustained impact post transition. A minimum score should be determined among key stakeholders and will vary by context. We do not yet understand if certain dimensions are more likely to lead to better outcomes post transition compared to other dimensions. Future applications of this tool, coupled with evaluation of solution impact post transition, will help illustrate whether certain dimension scores can better predict sustained solution impact at scale. Additionally, this assessment is just one of the many steps that are important to the transition process. Others include developing a clear solution description and transition strategy in partnership with funders and government partners. 

## Conclusion

Transitioning solution ownership to government enables greater likelihood that solutions will be sustained at scale and strengthen, rather than burden, health systems. The TRA is a flexible tool for NGOs, their government partners, and funders to make informed decisions for effectively transitioning solutions. This assessment can be applied to a variety of solutions across development sectors, uses accessible data collection methods, and uses consensus building among key stakeholders to generate actionable results. NGOs and their government partners can find and address transition challenges by measuring the seven dimensions of readiness, as well as adapt solution implementation and transition strategy if necessary.

While we are only beginning to use the TRA to assess our solutions, there is an urgent need for guidance among those who are following a similar path of government-owned solutions. For this reason, we are adding to the growing conversation about solution sustainability and transition by sharing access to a standardized tool to evaluate readiness for transition. We hope others can learn from our experiences testing this tool and can utilize the TRA and our recommendations to improve collaborative transition planning to achieve government-owned solutions that sustain impact at scale.

## Data availability

### Underlying data

No data is associated with this article.

### Extended data

FigShare: Transition Readiness Assessment: a tool for assessing readiness for integration of health solutions into government systems, https://doi.org/10.6084/m9.figshare.13158062.v1 ^16^.

This project contains the following extended data:

- Transition Readiness Assessment Tool
- TRA guideline document

Figshare: Transition Readiness Assessment Solution 1, https://doi.org/10.6084/m9.figshare.13158083.v1 ^19^.

Figshare: Transition Readiness Assessment Solution 2, https://doi.org/10.6084/m9.figshare.13158080.v1 ^20^.

Figshare: Interview Write Up Template for Accompanying TRA Government Interviews, https://doi.org/10.6084/m9.figshare.13158089.v1 ^18^.

Data are available under the terms of the Creative Commons Attribution 4.0 International license (CC-BY 4.0).

## Footnotes

^a^ VillageReach defines transition as the process of integrating a solution into existing public health care systems. Other terms used to describe this process are adoption, scaling up or institutionalization.
