# Supplementary material for: Integration of health solutions into government systems: a tool for assessing readiness
Source: Gates Open Res. 2020 Nov 19;4:172. [Version 1] doi: 10.12688/gatesopenres.13193.1 (PMC8236901; doi:10.12688/gatesopenres.13193.1)
Supplement: Peer review history

# Peer review: Reviewer response for version 1

**DOI:** 10.21956/gatesopenres.14399.r30768

**Published:** 2021-06-25

**Authors:** Neil Spicer

**Affiliations:** 1 Department of Global Health and Development, Faculty of Public Health and Policy, London School of Hygiene & Tropical Medicine, London, UK

The piece is a useful contribution and is worth indexing. I suggest the authors strengthen a few fairly minor issues:

- There is quite a lot more literature out there on ‘scale-up’ and ‘sustainability’ that looks at government adoption of donor-funded interventions – including frameworks and concepts. And so, the introduction underplays the amount already known of the subject, and how the current paper will contribute to that knowledge. So, I would suggest the authors acknowledge a wider pool of literature and tighten the justification for the paper’s addition/contribution.
- Is the study focused on a particular country – good to clarify that here?
- Figure 1 – needs a source ref if published previously?
- Section on the TRA - it might be helpful to clarify the relationship between the ‘transition to government’ framework and the TRA. Was the transition to government framework developed earlier by the same team and published in an earlier paper – and now this current paper is applying it via the TRA Excel based tool? A few words at the beginning of the section guiding the reader through would help a lot. And again, in which country was the work done?
- Methods - where did the work/data collection take place? And a bit more information is needed on the interviewing – who, selection criteria, where, etc.
- The modified nominal group approach – can the authors add a few words on how this technique was developed (perhaps add a reference if it is described elsewhere and say how the approach was modified?).
- In the key observations - it would help to remind the reader of what solution 1 and solution 2 are when discussing them in this section – and also in Table 2?

Does the article adequately reference differing views and opinions?

Partly

Are all factual statements correct, and are statements and arguments made adequately supported by citations?

Partly

Is the Open Letter written in accessible language?

Yes

Where applicable, are recommendations and next steps explained clearly for others to follow?

Yes

Is the rationale for the Open Letter provided in sufficient detail?

Yes

Reviewer Expertise:

Health policy analysis; qualitative methods; scale-up and sustainability of health interventions

I confirm that I have read this submission and believe that I have an appropriate level of expertise to confirm that it is of an acceptable scientific standard, however I have significant reservations, as outlined above.

# Peer review: Reviewer response for version 1

**DOI:** 10.21956/gatesopenres.14399.r30558

**Published:** 2021-04-16

**Authors:** Gilles Dussault

**Affiliations:** 1 Global Health and Tropical Medicine, Universidade Nova de Lisboa, Lisbon, Portugal

This Open Letter presents the Transition Readiness Assessment (TRA), a tool developed and tested by Village Reach with a view to encourage other development actors to try it and even improve it. The Letter is worth publishing in order to give visibility to a well-thought tool, that can usefully help plan transitioning in a rational manner. It offers a series of recommendations for future application; recommendations about when to use the TRA, e.g. at various stages of the planning of the transition and even as a monitoring tool after transitioning, is particularly relevant. The reader is invited to visit a dedicated website where a guideline document is available.

 I suggest that the authors add comments on the following points:

- The theoretical framework that structures the TRA gives equal value to its seven dimensions. Is there a way to modulate the weighing to reflect differences in the relative importance of each dimension? Also, is a low score on only one dimension enough to conclude that the solution is not ready for transition?
- The authors state “This assessment can be applied to a variety of solutions across development sectors”. What are examples of other solutions? Do some types of solutions present greater challenges for transition?
- Give information on the expertise required from national counterparts, and on time and financial resources needed to apply the TRA.
- What are the main facilitators/obstacles to the effective use of the TRA?

Does the article adequately reference differing views and opinions?

Yes

Are all factual statements correct, and are statements and arguments made adequately supported by citations?

Yes

Is the Open Letter written in accessible language?

Yes

Where applicable, are recommendations and next steps explained clearly for others to follow?

Yes

Is the rationale for the Open Letter provided in sufficient detail?

Yes

Reviewer Expertise:

health policy analysis, health workforce development

I confirm that I have read this submission and believe that I have an appropriate level of expertise to confirm that it is of an acceptable scientific standard.

# Peer review: Reviewer response for version 1

**DOI:** 10.21956/gatesopenres.14399.r30436

**Published:** 2021-03-24

**Authors:** Wilson Wong

**Affiliations:** 1 Department of Government and Public Administration, The Chinese University of Hong Kong, Hong Kong, Hong Kong

The article has provided a comprehensive and useful framework, Transition Readiness Assessment (TRA), for assessing the readiness of having a solution to be transited to government as a permanent and institutionalized measure. There is a total of seven dimensions in the framework (political, economic, social context; solution, design, resource availability; financial management; government strategy; government policy and regulations; and organizational management) which become a very comprehensive checklist for evaluating the strengths and weaknesses of a government in implementing and institutionalizing the solution. The main idea is to have it as a pre-testing and also ongoing exercise for facilitating dialogue and communication among major parties in seeking progress and improvement.

 While TRA is a very intelligent approach which certainly represents a major step forward in this area of research and practice, the current version of the article does have some blind spots and limitations which should be addressed in a revision.

 First of all, it has adopted a more state-led approach which assumes the transition of a solution from NGOs to government would be the ideal end state of the solution. However, while this can be one of the permanent arrangements, it is not sure whether it is possible and desirable to cover all cases and situations. Obviously, NGOs and other members of the civil society often have an indispensable role to play so that having a complete take-over by government may not represent the full spectrum of all scenarios. To resolve this, the article should spell out more clearly and explicitly the assumptions and conditions that need to be met for a complete takeover by government. In short, the authors should make it very clear under what conditions the TRA would be useful and applicable.

 Second, in the TRA, such as Figure 1, it is very good that political, economic and social contexts, are mentioned. Unfortunately, there are two problems here. There is no sufficient elaboration of the three contexts in the text on what they mean and how they are going to be evaluated in constructing the final score of the TRA. Besides, it does not seem to make sense to state that these contexts are not specific to a given solution and have no interaction with the integrated solution elements. It should not be difficult to think of situations in which the political context, such as a very closed and corrupted political system, can affect the policy and regulatory element and the organizational capacity of a government. These interactions and connections should be built into the revised TRA.

 Third, the role of civil society seems to be treated as only temporary and is replaceable as it is implied that the shift of the solution from civil society to government is the desirable end state. This line of argument is totally inconsistent with the trend of coproduction of public services and policies between state and society, and the major field of collaborative governance in the literature of public policy and administration. Most particularly, during the COVID-19 crisis, there is a bloom of theory-guided and empirical-based literature and studies discussing the important and critical role of civil society in combatting the pandemic. Links to some of the representative works are attached (for example: Wong, 2021 ^1^ and Cai *et al.*, 2021 ^2^).

 It is strongly recommended that the TRA should include the role of civil society in co-production both in the transition and the post-transition, such as how NGOs and citizens can play a visible and significant role in ensuring the positive attributes of public service such as transparency, openness, and accountability can be maintained in the implementation of the solution after it becomes government-owned.

Does the article adequately reference differing views and opinions?

Partly

Are all factual statements correct, and are statements and arguments made adequately supported by citations?

Partly

Is the Open Letter written in accessible language?

Yes

Where applicable, are recommendations and next steps explained clearly for others to follow?

Yes

Is the rationale for the Open Letter provided in sufficient detail?

Yes

Reviewer Expertise:

Technology Policy and Innovation; Comparative Public Policy

I confirm that I have read this submission and believe that I have an appropriate level of expertise to confirm that it is of an acceptable scientific standard, however I have significant reservations, as outlined above.

## References

[1] : When the state fails, bureaucrats and civil society step up: analysing policy capacity with political nexus triads in the policy responses of Hong Kong to COVID-19. *Journal of Asian Public Policy*.2021; 10.1080/17516234.2021.1894314 1-15 10.1080/17516234.2021.1894314

[2] : Civil Society Responses to the COVID-19 Pandemic: A Comparative Study of China, Japan, and South Korea. *The China Review*.2021;21(1) :107-137 Reference source
